# Evaluating PREDICT and developing outcome prediction models in early-onset breast cancer using data from Alberta, Canada

**DOI:** 10.1007/s10549-025-07654-1

**Published:** 2025-03-12

**Authors:** Robert B. Basmadjian, Yuan Xu, May Lynn Quan, Sasha Lupichuk, Winson Y. Cheung, Darren R. Brenner

**Affiliations:** 1 Department of Community Health Sciences, Cumming School of Medicine, University of Calgary, Calgary, AB Canada; 2 Department of Oncology, Cumming School of Medicine, University of Calgary, Calgary, AB Canada; 3 Department of Surgery, Cumming School of Medicine, University of Calgary, Calgary, AB Canada; 4 Department of Oncology and Community Health Sciences, Cumming School of Medicine, University of Calgary, Health Research Innovation Centre Room 2AA21, 3230 Hospital Dr NW, Calgary, AB T2N 4Z6 Canada

**Keywords:** Early-onset, Breast cancer, Outcome prediction, Machine learning, Decision making

## Abstract

**Introduction:**

Outcome prediction research in early-onset breast cancer (EoBC) is limited. This study evaluated the predictive performance of NHS PREDICT v2.1 and developed two prediction models for 5-year and 10-year all-cause mortality in a cohort of EoBC patients in Alberta, Canada.

**Methods:**

Adults < 40 years diagnosed with invasive breast cancer in Alberta, Canada from 2004 to 2020 were included. Patient data were entered into PREDICT v2.1 and mortality estimates at 5 and 10 years were extracted. Two prediction models were developed for all-cause mortality: multivariable Cox regression with LASSO penalization (LASSO Cox) and random survival forests (RSF). Internal validation of the developed models was performed using nested tenfold cross-validation repeated 200 times. Model performance was assessed using receiver operator characteristic and calibration curves for mortality at 5 and 10 years.

**Results:**

In total, 1827 patients with EoBC were eligible for inclusion. At 5 years, PREDICT had an area under the curve of 0.78 (95%CI 0.74–0.82) and overestimated mortality by 2.4% (95%CI 0.70–4.33) in the overall cohort. No differences in observed and predicted mortality by PREDICT were observed at 10 years. The LASSO Cox model showed better discrimination at 5 and 10 years than the RSF model, but both had poor calibration and underestimated mortality.

**Conclusion:**

PREDICT v2.1 tended to overestimate 5-year mortality in those with > 30% predicted risks and 10-year mortality in those with > 50% predicted risks for EoBC in Alberta, Canada. We did not identify additional models that would be clinically useful by applying machine learning. More follow-up data and emerging systemic treatment variables are required to study outcome prediction in modern cohorts.

**Supplementary Information:**

The online version contains supplementary material available at 10.1007/s10549-025-07654-1.

## Introduction

Breast cancer is the most common malignancy in Canadian women with an estimated 30,500 new cases in 2024 [1]. Approximately 4.5% of new cases are early-onset breast cancer (EoBC), defined as a diagnosis before 40 years of age [1]. In Alberta, Canada, (Surveillance & Reporting: The 2024 Report on Cancer Statistics in Alberta. Edmonton: Cancer Care AB, Alberta Health Services, 2024. https://www.albertahealthservices.ca/cancer/Page1774.aspx Access date: January 2025) the incidence rate of breast cancer begins to rise at age 25 and reaches 115 cases per 100,000 women in the 35-39 age group. Although diagnoses are uncommon, it is the most common cause of cancer death in this age group [2]. Patients with EoBC are more likely to have clinical and molecular features that are associated with worse survival than their older counterparts, including later stage disease and triple-negative breast cancers [3–5]. Further, EoBCs represent a minority of breast cancers and are consequentially underrepresented in randomized clinical trials of novel chemotherapies and targeted agents [6]. As such, robust evidence on optimal clinical management of EoBC is lacking.

The use of online decision-support tools to predict outcomes and determine the value of adjuvant systemic therapies in breast cancer has increased over the past decade [7, 8]. One of the most widely used web-based decision-support tools in breast cancer care is PREDICT [9]. It estimates overall survival (OS) at 5, 10, and 15 years after receiving surgery alone and the additional benefit of receiving the adjuvant systemic therapies. However, few women < 40 years were included in the sample from which PREDICT was trained and developed. Maishman et al. demonstrated subpar performance of PREDICT v1.2 in a cohort of 3000 patients < 40 years in the United Kingdom [10], which helped inform updated versions of PREDICT (v2.0-2.2) [11]. Since then, these updates have not been investigated in the early-onset setting.

One criticism levied against PREDICT is that it is built on a dated cohort that does not reflect the modern treatment era, particularly for women of younger ages. There remain concerns of overtreating EoBC patients with extensive surgery and chemotherapy based on age alone [6, 12, 13]. They are also more likely to experience disease- and treatment-related concerns unique to their age, including reproduction, sexuality, family responsibilities, and work productivity [12, 13]. Given PREDICT’s increasing use and endorsement in several practice guidelines, investigations on its performance in younger women are merited to ensure optimal clinical management. Further, these age-specific concerns represent the potential for new decision-support tools to help meet the tailored needs of this patient population. Since the publication of PREDICT, there has been considerable advancement, interest, and investment in machine learning approaches for outcome prediction in healthcare settings. Some pose machine learning as offering more flexible approaches to learning complex non-linear effects and high-order interactions between predictors [14]. However, these methods are rarely applied in EoBC, and it is unclear whether machine learning-based feature selection and model fitting could be beneficial in this context.

The current study assessed the predictive performance of PREDICT’s 5-year and 10-year all-cause mortality estimates in a real-world population of patients with EoBC in Alberta, Canada. This study also developed prediction models for all-cause mortality using regularized Cox regression and random survival forests, and performances at 5 and 10 years were compared to the PREDICT tool.

## Methods

This study reports the details of the development of prediction models in accordance with the transparent reporting of a multivariable prediction model for individual prognosis or diagnosis (TRIPOD) guidelines [15].

### Study population and data sources

This population-based, retrospective cohort included all women diagnosed with non-metastatic, invasive breast cancer aged ≥ 18 to < 40 years identified through the Alberta Cancer Registry from January 1st, 2004 to December 31st, 2020, with no malignancy 5 years prior to diagnosis. The variables of interest were merged from the registry, hospitalization discharge abstract database, national ambulatory care reporting system database, and vital statistics. Administrative end of follow-up was April 15th, 2022. Table 1 of the Supplemental Information includes further details on the Alberta Cancer Registry and all predictors, including their source dataset, original nature, and characterizations in PREDICT and our developed models.

### PREDICT v2.1

Survival estimates for PREDICT are derived from a competing risks Cox proportional hazards model stratified by estrogen receptor (ER) status. The output provides OS after receiving surgery alone and the additional benefit of receiving the selected adjuvant treatments. Variables and their characterizations in PREDICT can be found at https://breast.predict.nhs.uk/tool.

PREDICT was evaluated in all patients who underwent primary surgery with complete data corresponding to the tool’s variable characterizations, herein referred to as the PREDICT cohort. Each patient’s data were entered in PREDICT and the compliment of their 5- and 10-year OS estimates were extracted to obtain probabilities of 5- and 10-year all-cause mortality using the “nhs.predict” package in R. For all patients, Ki-67 was unknown as this variable was not present in our data. Method of detection was entered as “symptoms” for all patients as the < 40 years age group is not included in general population screening guidelines in Canada. Measured tumor size was not available, so tumor size was randomly imputed from a uniform distribution according to T-stage tumor size range (millimeters). Finally, use of bisphosphonates was entered as “no” as all patients were presumed premenopausal.

### Development of prediction models

Two prediction models for all-cause mortality were developed: (1) Multivariable Cox proportional hazards regression penalized with least absolute shrinkage and selection operator (LASSO) regularization, called the LASSO Cox model; and (2) random survival forests (RSF), called the RSF model. Models were developed in patients with complete data for all potential predictors of interest, mentioned below, and referred to as the development cohort. Data-driven approaches were used for feature selection in both models. For the LASSO Cox model, the penalization parameter (*λ*) was tuned to minimize the mean cross-validated error (1-Harrell’s C index) using tenfold cross-validation.

Feature selection for the RSF model was based on permutation importance measured by out-of-bag accuracy and occurred in a backwards elimination fashion, detailed elsewhere [16]. The number of trees was 1000. The number of randomly sampled predictors and tree depth used in the RSF model were tuned to minimizing the out-of-bag error (1-Harrell’s C index) using a multidimensional grid approach of possible values for these parameters.

The full list of variables that could be included as features in the final models included age (years), average neighborhood annual income [< $38,500, $38,500–$57,499, ≥ $57,500 (CAD)], treatment zone (academic, community), distance to closest treatment center (< 8.5 km, 8.5–26.5 km, > 26.5 km), tumor stage (T1, T2, T3, T4), number of positive lymph nodes (0, 1–3, ≥ 4), tumor grade (I, II, III), breast surgery [none, mastectomy, breast conservation surgery (BCS)], lymph node surgery (none, sentinel lymph node biopsy, axillary lymph node dissection), ER/progesterone receptor (PR)/human epidermal growth factor receptor-2 (HER2) status (positive, negative), initiation of hormone therapy (yes, no), ovarian function suppression (OFS) (no, oophorectomy, OFS), chemotherapy (yes, no), anti-HER2 therapy [trastuzumab or pertuzumab (yes, no)], and radiation (yes, no).

### Evaluating predictive performance

Predictive performance of the models was compared using discrimination and calibration measures. Discrimination was measured by time-dependent area under the receiver operating characteristic curve (AUC(*t*)) at 5 and 10 years [17]. Calibration was measured using *E*_max,_ the maximum difference between a smooth calibration curve and the diagonal line of perfect calibration [18]; integrated calibration index (ICI), the average difference between a smooth calibration curve and the diagonal line of perfect calibration [18]; and calibration slope and intercept. We also compared observed and predicted mortality using methods described by Engelhardt et al. [19] We used 1000 bootstrap resamples to create a distribution of differences between mean of the predicted mortality estimates and the observed mortality proportion for calculation of the 95% confidence interval, and bootstrap *P*-values were directly calculated from the distribution using the percentiles and simple sampling method [19]. Internal validation of the LASSO Cox and RSF model was performed with nested tenfold cross-validation repeated 200 times, where the inner folds were used for hyperparameter tuning and model fitting, and the outer folds were used to estimate performance metrics by taking the averages over the 2000 iterations. To estimate 95% confidence intervals (95%CI), 1000 bootstrap samples with replacement were drawn from the averaged metrics of the 200 cross-folds and the standard deviations of the corresponding bootstrap distributions were used to estimate standard error. Analyses were performed in RStudio. The “glmnet” package was used to tune and fit the LASSO Cox model, “randomForestSRC” was used to tune and fit the RSF model, “timeROC” was used to calculate AUC, and “rms” was used to generate calibration curves and metrics.

## Results

### Cohort characteristics

**Fig. 1:**
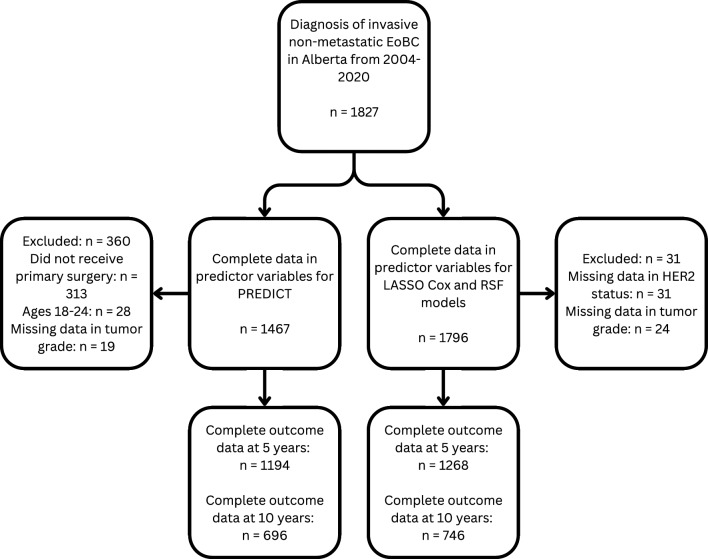
Inclusion and exclusion of individuals diagnosed with invasive non-metastatic breast cancer < 40 years of age in Alberta from 2004 to 2020. For the PREDICT cohort, individuals with ages 18–24 years, missing tumor grade data, or no primary surgery were excluded. For the development cohort, individuals with missing tumor grade and HER2 status data were excluded

In total, 1827 women < 40 years were diagnosed with invasive non-metastatic breast cancer in Alberta from January 1st, 2004, to December 31st, 2020. Of these, 1467 were included in the PREDICT cohort and 1796 were included in the development cohort. Figure 1 presents a flowchart of eligible individuals included in both cohorts, and the number of individuals with complete outcome data at 5 and 10 years. Characteristics of the development cohort are presented in Table 1. The median age of diagnosis [IQR] was 36 years [33–38] and majority (89.4%) were treated in academic facilities. 1376 (76.6%) and 563 (31.3%) patients were ER-positive and HER2-positive, respectively. Mastectomy was the preferred (47.2%) surgical procedure and 1277 (71.1%) received radiation. For systemic treatments, 1442 (80.3%) initiated chemotherapy, 365 (20.3%) initiated anti-HER2 therapy, 1098 (61.1%) initiated hormone therapy, and 343 (19.1%) initiated OFS therapy. Patient characteristics of the PREDICT cohort, according to variable characterizations of PREDICT, are presented in Table 2 of the Supplemental Information.

**Table 1:** Patient characteristics of the development cohort (*n* = 1796), which included all patients diagnosed with invasive non-metastatic breast cancer < 40 years of age with complete data in Alberta from 2004 to 2020

Characteristics	Total
	(*n* = 1796)
Age of diagnosis (years)	
Mean (SD)	34.7 (3.75)
Median [IQR]	36.0 [33.0, 38.0]
Treatment facility	
Academic	1606 (89.4%)
Rural	190 (10.6%)
Distance to closest treatment center (km)	
< 8.5	430 (23.9%)
8.5–26.5	905 (50.4%)
26.5+	461 (25.7%)
Average annual neighborhood income (CAD)	
< 38,500	452 (25.2%)
38,500–57499	905 (50.3%)
57,500+	440 (24.5%)
ER status	
Negative	420 (23.4%)
Positive	1376 (76.6%)
PR status	
Negative	663 (36.9%)
Positive	1133 (63.1%)
Her2 status	
Negative	1233 (68.7%)
Positive	563 (31.3%)
T stage	
T1	715 (39.8%)
T2	824 (45.9%)
T3	192 (10.7%)
T4	65 (3.6%)
Number of positive lymph nodes	
Zero	956 (53.2%)
1 to 3	608 (33.9%)
4+	232 (12.9%)
Grade	
I	162 (9.0%)
II	509 (28.3%)
III	1125 (62.6%)
Surgery type	
No surgery	308 (17.1%)
BCS	640 (35.6%)
Mastectomy	848 (47.2%)
Lymph node surgery type	
No surgery	308 (17.1%)
Sentinel lymph node biopsy	789 (43.9%)
Axillary lymph node dissection	699 (38.9%)
Chemotherapy	
No chemotherapy	354 (19.7%)
Chemotherapy	1442 (80.3%)
Anti-HER2 therapy	
No Anti-HER2 therapy	1431 (79.7%)
Anti-HER2 therapy	365 (20.3%)
Radiation therapy	
No radiation	519 (28.9%)
Radiation	1277 (71.1%)
Hormone therapy	
No hormone therapy	698 (38.9%)
Hormone therapy	1098 (61.1%)
Ovarian function suppression	
Neither	1153 (64.2%)
Oophorectomy	300 (16.7%)
OFS	343 (19.1%)

*BCS* breast-conserving surgery; *CAD* Canadian dollars; *ER* estrogen receptor; *HER2* human epidermal growth factor receptor 2; *IQR* interquartile range; *km* kilometers; *OFS* ovarian function suppression; *PR* progesterone receptor; *SD* standard deviation; *T stage* tumor stage

### Feature selection for LASSO Cox and RSF models

For the LASSO Cox model, the optimal penalization parameter (*λ*) was 0.026. The final predictors remaining after coefficient shrinkage were age, distance to closest treatment center, PR status, T stage, number of positive lymph nodes, breast surgery type, anti-HER2 therapy, radiation, hormone therapy, and OFS. For the RSF model, the optimal node size was 2. The 10 most important predictors selected were number of positive lymph nodes, ER status, T stage, PR status, chemotherapy, hormone therapy, grade, breast surgery type, radiation, and anti-HER2 therapy. Variable importance measures for included predictors are presented in Table 3 of the Supplemental Information.

### Model discrimination and calibration

**Fig. 2:**
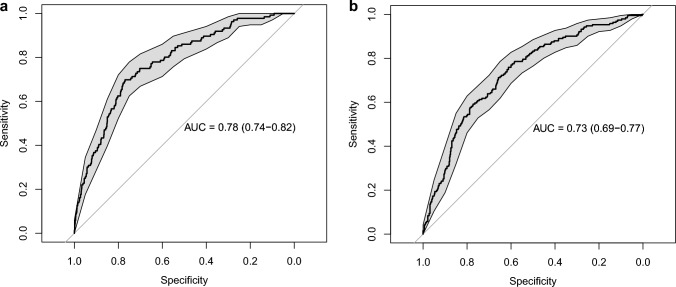
**a** Receiver operating characteristic curves for discriminatory accuracy of 5-year all-cause mortality for PREDICT v2.1. **b** Receiver operating characteristic curves for discriminatory accuracy of 10-year all-cause mortality for PREDICT v2.1

**Fig. 3:**
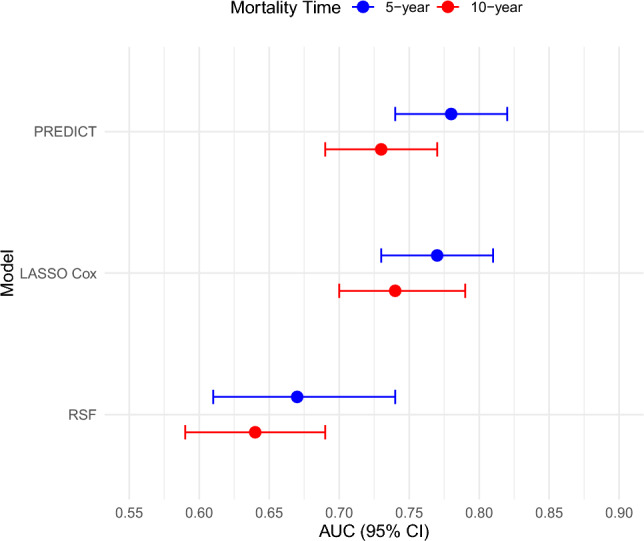
Area under the curve estimates for discriminatory accuracy of 5- and 10-year all-cause mortality for PREDICT v2.1, LASSO Cox, and random survival forest models

PREDICT’s AUC(t) was 0.78 (95%CI 0.74–0.82) for 5-year mortality (Fig. 2a) and 0.73 (95%CI 0.69–0.77) for 10-year mortality (Fig. 2b). The AUC values of the LASSO Cox and RSF models for 5- and 10-year mortality are presented in Fig. 3 and Table 2. The LASSO Cox model showed similar discrimination to PREDICT for 5- and 10-year mortality, but discrimination was poor for the RSF model at 5 years [AUC = 0.67 (95%CI 0.61–0.74)] and at 10 years [AUC = 0.64 (95%CI 0.59–0.69)].

**Table 2:** Discrimination and calibration measures of 5- and 10-year all-cause mortality probability estimates for PREDICT v2.1, LASSO Cox, and random survival forest models

Time	Measure (95%CI)	PREDICT v2.1	LASSO Cox model*	Random survival forest model*
5 years				
	AUC(*t*)	0.78 (0.74 to 0.82)	0.77 (0.73 to 0.81)	0.67 (0.61 to 0.74)
	Calibration slope	0.81 (0.65 to 0.97)	1.48 (1.18 to 1.71)	1.55 (1.24 to 1.98)
	Calibration intercept	− 0.27 (− 0.47 to − 0.07)	1.12 (0.54 to 1.63)	1.37 (0.83 to 1.91)
	ICI	0.03 (0.01 to 0.06)	0.14 (0.08 to 0.19)	0.16 (0.10 to 0.26)
	Emax	0.16 (0.11 to 0.23)	0.28 (0.15 to 0.38)	0.35 (0.26 to 0.48)
10 years				
	AUC(*t*)	0.73 (0.69 to 0.77)	0.74 (0.70 to 0.79)	0.64 (0.59 to 0.69)
	Calibration slope	0.56 (0.43 to 0.69)	1.36 (1.03 to 1.64)	1.49 (1.11 to 1.73)
	Calibration intercept	− 0.01 (− 0.21 to 0.18)	1.22 (0.68 to 1.68)	1.52 (1.04 to 1.86)
	ICI	0.05 (0.02 to 0.08)	0.12 (0.09 to 0.17)	0.16 (0.09 to 0.24)
	Emax	0.19 (0.14 to 0.25)	0.35 (0.25 to 0.43)	0.38 (0.29 to 0.51)

*AUC(t)* time-dependent area under the curve; *ICI* integrative calibration index; *LASSO Cox* multivariable proportional hazards Cox regression penalization with least absolute shrinkage and selection operator (LASSO) regularization

*Measures obtained using nested tenfold cross-validation repeated 200 times

**Fig. 4:**
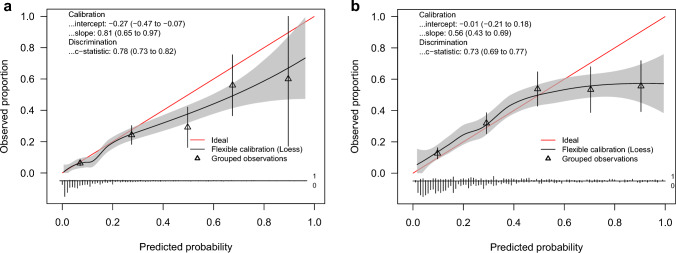
**a** Calibration curves of observed and predicted 5-year all-cause mortality for PREDICT v2.1. **b** Calibration curves of observed and predicted 10-year all-cause mortality for PREDICT v2.1

The calibration curves of PREDICT for 5- and 10-year mortality are presented in Fig. 4a and b, respectively. For 5-year mortality, the calibration slope and intercept were significantly less than 1 [slope = 0.81 (95%CI 0.65–0.97)] and 0 [intercept = − 0.27 (95%CI − 0.47 to − 0.47)], respectively (Table 2). In addition to the slope and intercept, the shape of the curve suggests overestimated 5-year mortality, particularly among those with estimates > 30% (Fig. 4a). For 10-year mortality, the slope was significantly less than 1 [slope = 0.56 (95%CI 0.43–0.69)] (Table 2). The shape the calibration curve shows overestimated 10-year mortality among those with estimates > 50% (Fig. 4b). The average (ICI) and largest (*E*_max_) differences between the calibration curve of PREDICT and ideal calibration line were similar for 5- and 10-year mortality. We also examined differences between average predicted mortality estimates from PREDICT and the observed mortality proportions (Table 3). In the overall cohort, PREDICT overestimated 5-year mortality by 2.40% (95%CI 0.70–4.33), but differences were not observed for 10-year mortality. In clinically relevant subgroups, PREDICT overestimated 5-year mortality in ER-positive [2.14% (95%CI 0.29–4.02)], HER2-positive [7.91% (95%CI 4.76–10.67)], grade III [4.03% (95%CI 1.71–6.44)], and T3 [9.57% (95%CI 1.71–16.97)] subgroups. Further, PREDICT underestimated 10-year mortality in T4 disease [− 24.4% (95%CI − 42.8 to − 5.53)]. A full table of event counts, AUCs, observed mortality proportions, and predicted mortality estimates from PREDICT by subgroup is presented in Table 4 of the Supplemental Information.

**Table 3:** Observed and predicted 5- and 10-year all-cause mortality for PREDICT v2.1, LASSO Cox, and random survival forest models

Time	Model	*N*	Alive	Dead	Observed (%)	Predicted (%)	Predicted-observed (95%CI)	*P*-value
5 years								
	PREDICT v2.1	1194	1038	136	11.39	13.79	2.40 (0.70 to 4.33)	0.004
	LASSO Cox	1268	1112	156	12.30	10.25	− 2.05 (− 3.80 to − 0.16)	0.013
	RSF	1268	1112	156	12.30	7.68	− 4.62 (− 6.21 to − 2.83)	< 0.0001
10 years								
	PREDICT v2.1	696	505	191	27.44	27.63	0.19 (− 2.80 to 3.38)	0.905
	LASSO Cox	746	532	214	28.69	17.71	− 10.98 (− 13.30 to − 8.50)	< 0.0001
	RSF	746	532	214	28.69	12.48	− 16.21 (− 20.03 to − 10.69)	< 0.0001

*LASSO Cox* multivariable proportional hazards Cox regression penalization with least absolute shrinkage and selection operator (LASSO) regularization; *RSF* random survival forests

The calibration metrics of the LASSO Cox and RSF models are shown in Table 2. For 5- and 10-mortality, the calibration slopes and intercepts of both models were greater than 1 and 0 suggesting overly modest and underestimated predictions. When compared with PREDICT, the ICI values of LASSO Cox and RSF models did not have overlapping confidence intervals for 5- or 10-year mortality. The same is true for *E*_max_ values, except for the LASSO Cox *E*_max_ value for 5-year mortality (Table 2).

## Discussion

To date, there is no decision-support tool for adjuvant treatment specific to patients < 40 years with breast cancer. It has become increasingly accepted that EoBC represents a patient population with poor prognosis and unique perspectives on treatment decisions [6]. PREDICT (v1) was initially developed on data from 5694 women who had surgery for invasive breast cancer in East Anglia from 1999 to 2003 and validated on external datasets of 5468 patients from the West Midlands and 3140 patients from British Columbia [20, 21]. Maishman et al. evaluated the prognostic performance of PREDICT v1.2 in a cohort of 3000 patients < 40 years in the United Kingdom. While 8- and 10-year survival predictions were reliable, 5-year survival was overestimated by 25% overall, 56% in ER-negative disease, and 13% in HER2-positive disease, and underestimated by 21% in ER-positive disease [10]. Engelhardt et al. evaluated 10-year mortality estimates from PREDICT v1.2 in a Dutch cohort of 2710 patients < 50 years and showed underestimated mortality (− 6.60%) among those < 35 years, and overestimated mortality in subgroups with a poor prognosis (e.g., node-positive, stage III, and T3) by 2.6% to 9.4% [19]. Additionally, PREDICT overestimated mortality in the HER2-negative subgroup by 2.2% [19]. PREDICT v2.0 is described to provide more accurate predictions in all age groups in three independent validation cohorts, in contrast to v1.2. Gray et al. performed an independent validation study in a Scottish cohort of 45,789 breast cancer patients. Among 456 women < 35 years, 5- and 10-year mortality were overestimated by 0.94% and 3.21%, although no statistical tests were conducted [22]. Another validation study in Dutch cohort of 8834 patients did not observe significant differences in predicted and observed mortality at 5 or 10 years, although point estimates demonstrate slight overestimation (2.2–2.7%) [23]. While both studies showed similar predicted and observed event rates, neither study present detailed calibration measures by age group.

Our study reports several calibration measures for PREDICT in the EoBC setting. The calibration intercept showed that the average predicted probability was greater than the overall event proportion at 5 years, but not at 10 years. Overestimation of 5-year mortality was observed in ER-positive, HER2-positive, grade III, and T3 disease. The slope, which evaluates the spread of the estimated risks, showed extreme 5- and 10-year predictions. Based on the shape of the calibration curves, this was true for 5-year predictions greater than 30%, and 10-year predictions greater than 50%. There may be several reasons for the overestimated predictions by PREDICT for high-risk individuals in this study. The first is that measured tumor size was not available in our data sources. While random numbers were imputed corresponding to the patients T stage, this would have resulted in misclassification. In PREDICT v2.0, the tumor size variable was refitted from discrete categories to measured size in millimeters [11], which may be more sensitive to errors in tumor size measures and thus prediction error. A second reason may be overrepresentation of higher-risk disease features in our EoBC cohort. Validation studies of PREDICT v2.0 observed overestimated 10-year mortality risk in HER2-positive, grade III, tumor size ≥ 30 mm, N2 or greater, and chemotherapy subgroups [22, 23]. These features were more frequent in our study and may have led to overestimated predictions in higher-risk individuals. Finally, miscalibration may be explained by a general limitation of outcome prediction based on dated cohorts, which is the inability to capture rapidly evolving treatments and practice guidelines. Chemotherapy benefits in PREDICT are derived from the 2012 Early Breast Cancer Trialists’ Collaborative Group meta-analysis, which does not compare taxane-based regimens with versus without anthracycline, dose-dense regimens, or the sequence of taxanes and anthracyclines [24]. Further PREDICT does not capture the benefits of OFS, post-mastectomy radiation, and anti-HER2 therapies beyond trastuzumab, all of which are pertinent to patients in contemporary cohorts. The evolution of treatment regimens in ER-positive and HER2-positive disease may explain why mortality is overestimated in these subgroups in particular. Beyond these, potential differences in the baseline hazards of mortality between the cohort used to develop PREDICT and our cohort may explain for miscalibration.

In lieu of our limitations in assessing PREDICT, we do not provide evidence that PREDICT should not be used in the EoBC population. Our findings show how missing predictors, like tumor size and Ki-67 index, influence PREDICT’s performance. This reflects that the population-level performance of PREDICT will likely be inferior to its performance on an individual level where clinicians have all data at hand to input. Therefore, we show how real-world data may be limited in assessing PREDICT’s performance.

The other primary aim of this study was to develop and compare data-driven modeling approaches for outcome prediction in the EoBC setting. We found that the LASSO Cox and RSF models provided less reliable predictions than PREDICT. This is shown by the ICI and *E*_max_ values, where the developed models displayed greater average and maximum prediction error. In other words, a large degree of uncertainty is expected for individual predictions, rendering these models ineffective in clinical practice. These findings point to the data being insufficient to support more complex data-driven approaches for outcome prediction in EoBC and may provide key insights for machine learning and clinical researchers in this setting. Our study included predictors that are routinely collected in standard breast cancer workup to reduce the burden and costs of collecting data. We applied LASSO penalization to Cox regression for variable selection to reduce the potential of overfitting, but calibration of the LASSO Cox model was still poor. While data-drive methods may assist with feature reduction, some excluded variables will be considered in the clinical setting. Expert opinion is still important and should be compared against. Subject knowledge is viewed as a useful technique to restrict the number of candidate predictors and increase the robustness and validity of prediction models, particularly in smaller samples [25–27]. Given that the EoBC population is small, expertise is vital to ensure clinical needs are being met. In particular, investigation of the predictive role of breast surgery type is merited given rising mastectomy rates despite emerging oncoplastic techniques for BCS procedures [28]. Additionally, OFS was recently implemented in real-world practice for adjuvant treatment of premenopausal hormone receptor-positive breast cancer. Fertility concerns in this age group impact the decision to receive OFS, despite survival benefits [29]. There is a pressing need to characterize the predictive potential of OFS to better communicate outcome risk and inform decision making.

Another important insight is that although random survival forests offer more flexible learning algorithms, they require large amounts of data. With only 18 predictors, it is likely that the sample size and dimensionality of the development cohort was insufficient, resulting in poor performance in testing data. Further, random survival forests use log-rank splitting when building decision trees, which may not be appropriate for all predictors. The additive and semi-parametric nature of Cox regression appears to be the most suitable for EoBC outcome prediction using routinely available data, as seen by PREDICT’s performance. Population-based administrative data and genomic databases contain a breadth of potential variables to improve prediction. However, models with many predictors, or predictors that are timely and costly to collect, may have limited uptake, demonstrating the need for collaboration between clinicians and model developers to build models that are accurate and easy to implement. The cost of collecting additional data to support complex learning algorithms is questionable. Others have compared Cox regression with RSF for outcome prediction in breast cancer with similar predictors to ours, yielding c-indexes of 0.60–0.70 for both model types [30, 31]. Even studies with large integrated genomic data did not demonstrate superior performance of RSF to Cox models [32].

A major strength of this study is that the data sources capture nearly all (> 93%) breast cancer cases in Alberta, Canada, resulting in a larger sample size of EoBC patients than what is observed in < 40 year subgroups of other validation studies. These findings may be inferred to other EoBC patients in this province and potentially others, because of the universal health system and similar patterns of care, and the similarities in administrative data between provinces in Canada. However, several limitations should be noted. First, no independent cohort was available for external validation of the LASSO Cox and RSF models so their performance in independent data is unknown. Second, several potentially relevant prognostic variables were not available for analysis, including body mass index, measured tumor size, tumor-infiltrating lymphocytes, p53 status, Ki-67 status, *BRCA1/2* status, race and ethnicity, and other social determinants of health. Finally, while this study attempted to consider treatments relevant to the EoBC setting, like OFS, their recent implementation reflects a common issue in long-term prediction of contemporary cohorts. Patients with more recent diagnoses consequently have shorter follow-up. Therefore, more follow-up data are required to further study PREDICT, the LASSO Cox, and RSF models in modern cohorts.

## Conclusion

Outcome prediction research in EoBC is limited. While modern versions of PREDICT show better predictive performance in patients < 40 years than previous versions, this tool does not reflect all locoregional and adjuvant treatment options specific to this age group. Decision-aid tools focused on the needs of younger breast cancer patients should become a research priority. The application of machine learning approaches did not improve outcome prediction compared with existing tools like PREDICT but predictors specific to EoBC should be investigated to support decision making in this setting.

## Supplementary Information

Below is the link to the electronic supplementary material.

Supplementary file1 (DOCX 31 KB)

## Data Availability

The data that support the findings of this study were used under license and ethics approval for this study and are not publicly available. Data may be made available upon reasonable request to the authors.

## References

[1] Brenner DR, Gillis J, Demers AA et al (2024) Projected estimates of cancer in Canada in 2024. Can Med Assoc J 196:E615–E623. 10.1503/cmaj.212097 38740416 10.1503/cmaj.240095 PMC11090635

[2] Siegel RL, Giaquinto AN, Jemal A (2024) Cancer statistics, 2024. CA Cancer J Clin 74:12–49. 10.3322/caac.21820 38230766 10.3322/caac.21820

[3] Anders CK, Johnson R, Litton J et al (2009) Breast cancer before age 40 years. Semin Oncol 36:237–249. 10.1053/j.seminoncol.2009.03.001 19460581 10.1053/j.seminoncol.2009.03.001 PMC2894028

[4] Azim HA Jr, Partridge AH (2014) Biology of breast cancer in young women. Breast Cancer Res 16:427–427. 10.1186/s13058-014-0427-5 25436920 10.1186/s13058-014-0427-5 PMC4303229

[5] Narod SA (2012) Breast cancer in young women. Nat Rev Clin Oncol 9:460–470. 10.1038/nrclinonc.2012.102 22733233 10.1038/nrclinonc.2012.102

[6] Paluch-Shimon S, Cardoso F, Partridge AH et al (2022) ESO-ESMO fifth international consensus guidelines for breast cancer in young women (BCY5). Ann Oncol 33:1097–1118. 10.1016/j.annonc.2022.07.007 35934170 10.1016/j.annonc.2022.07.007

[7] Engelhardt EG, Garvelink MM, de Haes JCJM et al (2013) Predicting and communicating the risk of recurrence and death in women with early-stage breast cancer: a systematic review of risk prediction models. J Clin Oncol 32:238–250. 10.1200/JCO.2013.50.3417 24344212 10.1200/JCO.2013.50.3417

[8] Shachar SS, Muss HB (2016) Internet tools to enhance breast cancer care. npj Breast Cancer 2:16011. 10.1038/npjbcancer.2016.11 28721377 10.1038/npjbcancer.2016.11 PMC5515323

[9] Cambridge Breast Unit University of Cambridge Department of Oncology and the UK’s Eastern Cancer Information and Registration Centre (ECRIC): Predict Breast Cancer. Winton Centre for Risk & Evidence Communication. https://breast.predict.nhs.uk/. Accessed Jan 2023

[10] Maishman T, Copson E, Stanton L et al (2015) An evaluation of the prognostic model PREDICT using the POSH cohort of women aged ≤40 years at breast cancer diagnosis. Br J Cancer 112:983–991. 10.1038/bjc.2015.57 25675148 10.1038/bjc.2015.57 PMC4366898

[11] Candido dos Reis FJ, Wishart GC, Dicks EM et al (2017) An updated PREDICT breast cancer prognostication and treatment benefit prediction model with independent validation. Breast Cancer Res 19:58. 10.1186/s13058-017-0852-3 28532503 10.1186/s13058-017-0852-3 PMC5440946

[12] Sella T, Snow C, Freeman H et al (2021) Young, empowered and strong: a web-based education and supportive care intervention for young women with breast cancer across the care continuum. JCO Clin Cancer Inform. 10.1200/CCI.21.00067 34473546 10.1200/CCI.21.00067

[13] Tesch ME, Partridge AH (2022) Treatment of breast cancer in young adults. Am Soc Clin Oncol Educ Book 42:795–806. 10.1200/EDBK_360970 10.1200/EDBK_360970 35580291

[14] Clift AK, Dodwell D, Lord S et al (2023) Development and internal-external validation of statistical and machine learning models for breast cancer prognostication: cohort study. BMJ 381:e073800. 10.1136/bmj-2022-073800 37164379 10.1136/bmj-2022-073800 PMC10170264

[15] Moons KG, Altman DG, Reitsma JB et al (2015) Transparent Reporting of a multivariable prediction model for Individual Prognosis or Diagnosis (TRIPOD): explanation and elaboration. Ann Intern Med 162:W1-73. 10.7326/m14-0698 25560730 10.7326/M14-0698

[16] Basmadjian RB, Kong S, Boyne DJ et al (2022) Developing a prediction model for pathologic complete response following neoadjuvant chemotherapy in breast cancer: a comparison of model building approaches. JCO Clin Cancer Inform 6:e2100055. 10.1200/cci.21.00055 35148170 10.1200/CCI.21.00055 PMC8846388

[17] Blanche P, Dartigues J-F, Jacqmin-Gadda H (2013) Estimating and comparing time-dependent areas under receiver operating characteristic curves for censored event times with competing risks. Stat Med 32:5381–5397. 10.1002/sim.5958 24027076 10.1002/sim.5958

[18] Austin PC, Steyerberg EW (2019) The Integrated Calibration Index (ICI) and related metrics for quantifying the calibration of logistic regression models. Stat Med 38:4051–4065. 10.1002/sim.8281 31270850 10.1002/sim.8281 PMC6771733

[19] Engelhardt EG, van den Broek AJ, Linn SC et al (2017) Accuracy of the online prognostication tools PREDICT and adjuvant! For early-stage breast cancer patients younger than 50 years. Eur J Cancer 78:37–44. 10.1016/j.ejca.2017.03.015 28412587 10.1016/j.ejca.2017.03.015

[20] Wishart GC, Azzato EM, Greenberg DC et al (2010) PREDICT: a new UK prognostic model that predicts survival following surgery for invasive breast cancer. Breast Cancer Res 12:R1. 10.1186/bcr2464 20053270 10.1186/bcr2464 PMC2880419

[21] Wishart GC, Bajdik CD, Azzato EM et al (2011) A population-based validation of the prognostic model PREDICT for early breast cancer. Eur J Surg Oncol 37:411–417. 10.1016/j.ejso.2011.02.001 21371853 10.1016/j.ejso.2011.02.001

[22] Gray E, Marti J, Brewster DH et al (2018) Independent validation of the PREDICT breast cancer prognosis prediction tool in 45,789 patients using Scottish Cancer Registry data. Br J Cancer 119:808–814. 10.1038/s41416-018-0256-x 30220705 10.1038/s41416-018-0256-x PMC6189179

[23] van Maaren MC, van Steenbeek CD, Pharoah PDP et al (2017) Validation of the online prediction tool PREDICT v. 2.0 in the Dutch breast cancer population. Eur J Cancer 86:364–372. 10.1016/j.ejca.2017.09.031 29100191 10.1016/j.ejca.2017.09.031

[24] Early Breast Cancer Trialists’ Collaborative Group (2012) Comparisons between different polychemotherapy regimens for early breast cancer: meta-analyses of long-term outcome among 100,000 women in 123 randomised trials. The Lancet 379:432–444. 10.1016/S0140-6736(11)61625-5 10.1016/S0140-6736(11)61625-5 PMC3273723 22152853

[25] Steyerberg EW (2009) Clinical prediction models: a practical approach to development, validation, and updating—chapters 4 & 5. Springer, New York

[26] Steyerberg EW (2009) Clinical prediction models: a practical approach to development, validation, and updating—chapters 15–17. Springer, New York

[27] Steyerberg EW, Moons KG, van der Windt DA et al (2013) Prognosis Research Strategy (PROGRESS) 3: prognostic model research. PLoS Med 10:e1001381. 10.1371/journal.pmed.1001381 23393430 10.1371/journal.pmed.1001381 PMC3564751

[28] Findlay-Shirras L, Lima I, Smith G et al (2021) Canada follows the US in the rise of bilateral mastectomies for unilateral breast cancer: a 23-year population cohort study. Breast Cancer Res Treat 185:517–525. 10.1007/s10549-020-05965-z 33128192 10.1007/s10549-020-05965-z

[29] Francis PA, Pagani O, Fleming GF et al (2018) Tailoring adjuvant endocrine therapy for premenopausal breast cancer. N Engl J Med 379:122–137. 10.1056/NEJMoa1803164 29863451 10.1056/NEJMoa1803164 PMC6193457

[30] Kurt Omurlu I, Ture M, Tokatli F (2009) The comparisons of random survival forests and Cox regression analysis with simulation and an application related to breast cancer. Expert Syst Appl 36:8582–8588. 10.1016/j.eswa.2008.10.023

[31] Nicolò C, Périer C, Prague M et al (2020) Machine learning and mechanistic modeling for prediction of metastatic relapse in early-stage breast cancer. JCO Clin Cancer Inform. 10.1200/CCI.19.00133 32213092 10.1200/CCI.19.00133

[32] Yousefi S, Amrollahi F, Amgad M et al (2017) Predicting clinical outcomes from large scale cancer genomic profiles with deep survival models. Sci Rep 7:11707. 10.1038/s41598-017-11817-6 28916782 10.1038/s41598-017-11817-6 PMC5601479

