# Supplementary material for: Evaluating PREDICT and developing outcome prediction models in early-onset breast cancer using data from Alberta, Canada
Source: Breast Cancer Res Treat. 2025 Mar 12;211(2):399–408. doi: 10.1007/s10549-025-07654-1 (PMC12006220; doi:10.1007/s10549-025-07654-1)
Supplement: Supplementary file 1 — Supplementary file1 (DOCX 31 KB) [file 10549_2025_7654_MOESM1_ESM.docx]

**SUPPLEMENTAL INFORMATION**

**LIST OF TABLES AND FIGURES**

Table 1. The source, original nature, and characterization of predictor variables used in this study.

Table 2. Patient characteristics of the PREDICT cohort (n=1467), which included all patients diagnosed with invasive breast cancer <40 years of age and underwent primary surgery in Alberta from 2004 to 2020.

Table 3. Variable importance measures for 10 selected features in the random survival forest model, which was calculated by the change in out-of-bag accuracy following feature permutation.

Table 4. Observed and predicted 5- and 10-year all-cause mortality for PREDICT v2.1 in clinically relevant subgroups.

**Additional details on the data used**

The Alberta Cancer Registry (ACR), established in 1942, is legally mandated to collect and maintain records of all cancer diagnoses and deaths in the province. It gathers data from 17 cancer centers—comprising two tertiary, four regional, and eleven community centers—serving Alberta’s 4.5 million residents. ACR staff undergo specialized training to collect and interpret cancer data according to national and international coding standards, ensuring high-quality and comparable records.

The registry plays a crucial role in cancer research and planning, supporting efforts in prevention, early detection, and improved patient outcomes. Certified by the North American Association of Central Cancer Registries (NAACCR), the ACR has consistently maintained the highest Gold Certification status since 2002—the only Canadian registry to do so—based on its data completeness, timeliness, and quality.

The ACR collects various types of data, including demographic details (age, sex at birth, date of birth), tumor characteristics (diagnosis date and method, cancer type, stage, and tumor features), treatment information (treatment dates, types, and agents used), and mortality details (death date, location, and cause). Additionally, it can be linked with Canadian Census data for neighborhood-level insights using postal codes. The registry may also integrate with hospitalization discharge records and national ambulatory care databases, leveraging ICD-10 codes to enhance health and procedural data.

Table 1. The source, original nature, and characterization of predictor variables used in this study.

| **Predictor Variable** | **Source dataset** | **Original nature in data** | **Characterization in PREDICT v2.1** | **Characterization in development of LASSO Cox and RSF models** |
| --- | --- | --- | --- | --- |
| Age | ACR | Continuous (years) | Continuous (years) | Categorical: <35, 35-39 years |
| Treatment facility | ACR | Categorical - Calgary, Central, Edmonton, North, South | NA | Academic = Calgary, Edmonton, Community = Central, North, South |
| Distance to closest treatment centre | ACR | Continuous (km) | NA | Categorical: <8.5km, 8.5-26.5km, >26.5km |
| Average neighbourhood annual income | ACR | Continuous (CAD) | NA | Categorical: <38,500, 38,500- |
| Estrogen receptor status | ACR | Categorical - NAACCR SSF/SSDI coding: Positive, Negative, Unknown | Same as original nature | Same as original nature |
| Progesterone receptor status | ACR | Categorical - NAACCR SSF/SSDI coding: Positive, Negative, Unknown | Same as original nature | Same as original nature |
| HER2 status | ACR | Categorical - NAACCR SSF/SSDI coding: Positive, Negative, Unknown | Same as original nature | Same as original nature |
| Tumor stage | ACR | Categorical - AJCC TNM staging: T1, T1a, T1b, T1c, T1mi, T2, T3, T4 | NA | T1 = T1, T1a, T1b, T1c, otherwise same as original nature |
| Tumor size | NA | NA | Random imputation from uniform distribution based on AJCC T stage: T1mi (0.01-1.00mm), T1a (1.01-5.00mm), T1b (5.01-10.00mm), T1c (10.01-20.00mm), T2 (20.01-50.00mm), T3 (50.01-100.00), T4 (0.01-100.00mm) | NA |
| Nodal stage | ACR | Categorical - AJCC TNM staging: N0, N1, N1a, N1b, N1c, N2, N2a, N2b, N3, N3a, N3b | NA | NA |
| Number of positive lymph nodes | ACR | Count (whole integer of positive lymph nodes) | Same as original nature | Categorical: 0, 1-3, 4+ |
| Tumor grade | ACR | Categorical - AJCC TNM staging: Low, intermediate, high | Low = I, intermediate = II, high = III | Low = I, intermediate = II, high = III |
| Breast surgery type | DAD/NACRS | ICD-10 codes procedural codes | NA | No surgery = did not have surgical procedure date, BCS = 1YM87, 1YM88 , Mastectomy = 1YM89, 1YM90, 1YM91, 1YM92 |
| Lymph node surgery type | DAD/NACRS | ICD-10 codes procedural codes | NA | No surgery = did not have surgical procedure date, SLNB = 2MD87, 1MD87, ALND = 1MD89, 1YM91 |
| Radiation therapy | ACR | Primary treatment start date and fractions received | NA | No radiation = did not have primary treatment date, Radiation = had primary treatment date |
| Chemotherapy | ACR | Primary treatment start date and agent received | No chemotherapy = did not have primary treatment date or did not initiate anthracycline or taxane-based regimen, 2nd gen = anthracyclin with no taxane, 3rd gen = taxane with or without anthracyclin, high-dose anthracyclin | No chemotherapy = did not have primary treatment date, Chemotherapy = initiation of at least one agent chemotherapy agent of any kind |
| Anti-HER2 therapy | ACR | Primary treatment start date and agent received | No anti-HER2 therapy = did not have primary treatment date, Anti-HER2 therapy = initiation of trastuzumab or pertuzumab | No anti-HER2 therapy = did not have primary treatment data, Anti-HER2 therapy = initiation of trastuzumab or pertuzumab |
| Hormone therapy | ACR | Primary treatment start date and agent received | No hormone therapy = did not have primary treatment date, Hormone therapy = initiation of tamoxifen or aromatase inhibitor | No hormone therapy = did not have primary treatment date, Hormone therapy = initiation of tamoxifen or aromatase inhibitor |
| Ovarian function suppression therapy | ACR, DAD/NACRS | Primary treatment start date and agent received, ICD-10 codes for procedural codes | NA | No OFS = did not have primary treatment data and did not undergo oophorectomy, OFS agent = initiation of GnRH agonist, Oophorectomy = Z907, Z4002, 1RB87, 1RB89, 1RD89 |

Abbreviations: ACR = Alberta Cancer Registry; AJCC = American Joint Committee on Cancer; DAD = discharge abstract database; HER2 = human epidermal growth factor receptor 2; ICD = Internation Classification of Diseases; NA = not applicable; NAACCR = North American Association of Central Cancer Registries; NACRS = national ambulatory care reporting system; SSDI = site-specific data item; SSF = site-specific factor; TNM = tumor, node, metastasis.

Table 2. Patient characteristics for the PREDICT cohort (n=1467), which included all patients diagnosed with invasive breast cancer <40 years of age and underwent primary surgery in Alberta from 2004 to 2020. Characteristics are expressed in accordance to PREDICT.

|  | **Total** |
| --- | --- |
| **Characteristics** | **(N=1467)** |
| **Age of diagnosis** |  |
| Mean (SD) | 34.9 (3.50) |
| Median [IQR] | 36.0 [33.0, 38.0] |
| **Postmenopausal?** |  |
| Yes | 0 |
| No | 1467 (100%) |
| Unknown | 0 |
| **Detected by** |  |
| Screening | 0 |
| Symptoms | 1467 (100%) |
| **ER status** |  |
| Negative | 349 (23.8%) |
| Positive | 1118 (76.2%) |
| **Her2 status** |  |
| Negative | 973 (66.3%) |
| Positive | 488 (33.3%) |
| Unknown | 6 (0.4%) |
| **Tumor size (mm)** |  |
| Mean (SD) | 26.5 (20.9) |
| Median [IQR] | 25.9 [0.0100, 75.0] |
| **Tumor grade** |  |
| I | 97 (6.6%) |
| II | 422 (28.8%) |
| III | 948 (64.6%) |
| **Positive nodes** |  |
| Mean (SD) | 1.72 (3.80) |
| Median [IQR] | 0 [0, 55.0] |
| **Ki-67 status** |  |
| Positive | 0 |
| Negative | 0 |
| Unknown | 1467 (100%) |
| **Chemotherapy** |  |
| None | 647 (44.1%) |
| 2nd gen | 114 (7.8%) |
| 3rd gen | 706 (48.1%) |
| **Hormone therapy** |  |
| No | 585 (39.9%) |
| Yes | 882 (60.1%) |
| **Trastuzumab** |  |
| No | 1146 (78.1%) |
| Yes | 321 (21.9%) |
| **Bisphosphonates** |  |
| No | 1467 (100%) |
| Yes | 0 |

Abbreviations: ER = estrogen receptor; HER2 = human epidermal growth factor receptor 2; mm = millimetres; IQR = interquartile range; PR = progesterone receptor; SD = standard deviation.

Table 3. Variable importance measures for 10 selected features in the random survival forest model, which was calculated by the change in out-of-bag accuracy following feature permutation.

| **Feature** | **Variable Importance** |
| --- | --- |
| Number of positive lymph nodes | 0.041 |
| ER status | 0.033 |
| T stage | 0.028 |
| PR status | 0.016 |
| Chemotherapy | 0.012 |
| Hormonal therapy | 0.008 |
| Tumor grade | 0.004 |
| Breast surgery type | 0.0007 |
| Radiation therapy | 0.00028 |
| Anti-HER2 therapy | 0.00017 |

Abbreviations: ER = estrogen receptor; HER2 = human epidermal growth factor receptor 2; PR = progesterone receptor; T stage = tumor stage.

Table 4. Observed and predicted 5- and 10-year all-cause mortality for PREDICT v2.1 in clinically relevant subgroups.

| **Predictor** | **Categories** | **N** | **Alive** | **Dead** | **AUC (95% CI)** | **Observed (%)** | **Predicted (%)** | **Predicted-Observed (95% CI)** | **P-value** |
| --- | --- | --- | --- | --- | --- | --- | --- | --- | --- |
| Age | <35 | 454 | 394 | 60 | 0.78 (0.72-0.84) | 13.22 | 16.22 | 3.01 (-0.28 - 5.87) | 0.053 |
|  | 35-39 | 740 | 664 | 76 | 0.77 (0.73-0.81) | 10.27 | 12.3 | 2.03 (-0.19 - 4.11) | 0.065 |
| ER status |  |  |  |  |  |  |  |  |  |
|  | Negative | 262 | 207 | 55 | 0.77 (0.70-0.85 | 20.99 | 22.83 | 1.84 (-2.69 - 6.05) | 0.419 |
|  | Positive | 904 | 824 | 80 | 0.76 (0.71-0.81) | 8.85 | 10.99 | 2.14 (0.29 - 4.02) | 0.02 |
| HER2 status |  |  |  |  |  |  |  |  |  |
|  | Negative | 553 | 478 | 75 | 0.79 (0.73-0.85) | 13.56 | 11.86 | `-1.71 (-4.24 - 0.9) | 0.21 |
|  | Positive | 280 | 259 | 21 | 0.74 (0.64-0.85) | 7.5 | 15.41 | 7.91 (4.76 - 10.67) | <0.0001 |
| Grade |  |  |  |  |  |  |  |  |  |
|  | I | 68 | 65 | 3 | 0.90 (0.74-1.00) | 4.41 | 2.31 | `-2.11 (-7.56 - 2.16) | 0.394 |
|  | II | 330 | 315 | 15 | 0.85 (0.73-0.97) | 4.55 | 7.02 | 2.47 (0.43 - 4.29) | 0.008 |
|  | III | 774 | 668 | 106 | 0.75 (0.70-0.80) | 13.7 | 17.72 | 4.03 (1.71 - 6.44) | <0.0001 |
| Tumor stage |  |  |  |  |  |  |  |  |  |
|  | T1 | 478 | 459 | 19 | 0.78 (0.66-0.89) | 3.97 | 4.48 | 0.50 (-1.13 - 2.08) | 0.563 |
|  | T2 | 545 | 461 | 78 | 0.73 (0.67-0.80) | 14.31 | 17.09 | 2.78 (0.08-5.48) | 0.043 |
|  | T3 | 126 | 98 | 28 | 0.58 (0.46-0.71) | 22.22 | 31.79 | 9.57 (1.71-16.97) | 0.009 |
|  | T4 | 45 | 34 | 11 | 0.68 (0.46-0.90) | 24.44 | 22.31 | `-2.13 (-14.75 - 8.87) | 0.745 |
|  |  |  |  |  |  |  |  |  |  |
| Age | <35 | 258 | 176 | 82 | 0.71 (0.64 - 0.77) | 31.78 | 32.33 | 0.54 (-5.35 - 5.61) | 0.841 |
|  | 35-39 | 438 | 329 | 109 | 0.75 (0.69 - 0.80) | 24.89 | 24.86 | `-0.02 (-3.93 - 3.92) | 0.991 |
| ER status |  |  |  |  |  |  |  |  |  |
|  | Negative | 179 | 120 | 59 | 0.77 (0.70 - 0.85) | 32.96 | 31.91 | `-1.05 (-6.82 - 4.94) | 0.729 |
|  | Positive | 501 | 370 | 131 | 0.72 (0.67 - 0.77) | 26.15 | 26.16 | 0.01 (-3.92 - 3.84) | 0.996 |
| HER2 status |  |  |  |  |  |  |  |  |  |
|  | Negative | 206 | 113 | 93 | 0.80 (0.74 - 0.86) | 45.15 | 40.57 | `-5.61 (-11.83 - 2.7) | 0.223 |
|  | Positive | 139 | 102 | 37 | 0.68 (0.59-0.78) | 26.62 | 30.38 | 3.76 (-3.69 - 10.87) | 0.305 |
| Grade |  |  |  |  |  |  |  |  |  |
|  | I | 39 | 35 | 4 | 0.91 (0.78 - 1.00) | 10.26 | 6.67 | `-3.58 (-13.61 - 4.24) | 0.421 |
|  | II | 191 | 158 | 33 | 0.77 (0.68-0.87) | 17.28 | 15.5 | 1.78 (-6.65-3.31) | 0.502 |
|  | III | 454 | 312 | 142 | 0.71 (0.66-0.76) | 31.28 | 34.62 | 3.34 (-0.77 - 7.60) | 0.116 |
| Tumor stage |  |  |  |  |  |  |  |  |  |
|  | T1 | 272 | 239 | 33 | 0.64 (0.53-0.75) | 12.13 | 9.97 | `-2.17 (-6.31 - 1.47) | 0.295 |
|  | T2 | 321 | 217 | 104 | 0.69 (0.63-0.75) | 32.4 | 34.71 | 2.31 (-2.75 - 7.24) | 0.364 |
|  | T3 | 76 | 39 | 37 | 0.52 (0.38-0.65) | 48.68 | 57.04 | 8.35 (-3.34-21.72) | 0.192 |
|  | T4 | 27 | 10 | 17 | 0.59 (0.35-0.82) | 62.96 | 38.57 | `-24.4 (-42.8 - -5.53) | 0.012 |
